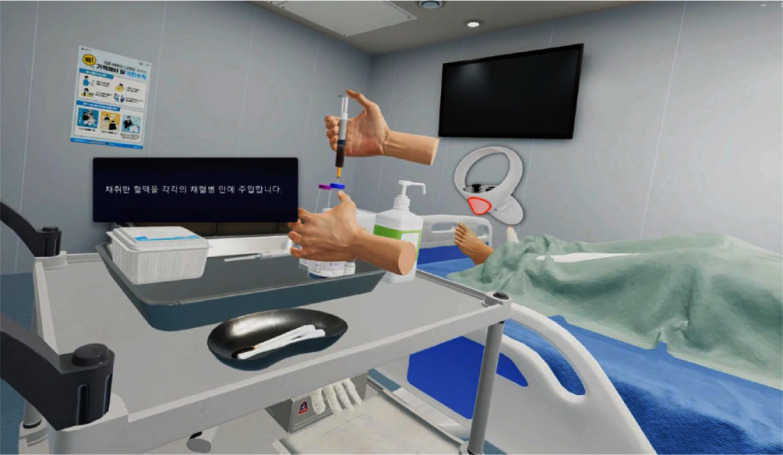# The Effectiveness of Virtual Reality-Based Multidrug-Resistant Organisms Infection Control Education for Nursing Students

**DOI:** 10.1017/ash.2025.427

**Published:** 2025-09-24

**Authors:** Kyungmi Kim, Jeong Sil Choi

## Abstract

**Purpose:** This study aimed to verify the effectiveness of a virtual reality (VR)-based multidrug-resistant organisms (MDROs) infection control education program for nursing students. **Methods:** This study is quasi-experimental with a nonequivalent control group pretest-posttest design. The subjects were 56 nursing students (28 in the experimental and 28 in the control group). A VR education program on infection control for MDROs was applied to the experimental group. The effectiveness of the education was assessed using a questionnaire. **Results:** The experimental and control groups had no statistically significant difference in the knowledge of MDROs infection control, performance confidence, and self-efficacy before and after the VR-based education. The difference in the knowledge of MDROs infection control between the experimental group before and after the VR education was 9.08±7.50, and the control group was 6.12±16.69. The difference value between the two groups was statistically significant (p = .036). The difference in performance confidence was 0.32±0.38 points in the experimental group and 0.27±0.52 points in the control group, and there was no statistically significant difference between the two groups (p = .073). The difference value of self-efficacy was 0.43±0.39 points in the experimental group and -0.23±0.71 points in the control group, and there was a statistically significant difference between the two groups **Conclusion:** This study found that This study found that VR-based infection control education can help acquire knowledge and self-efficacy in MDROs infection control.

**Figure:**
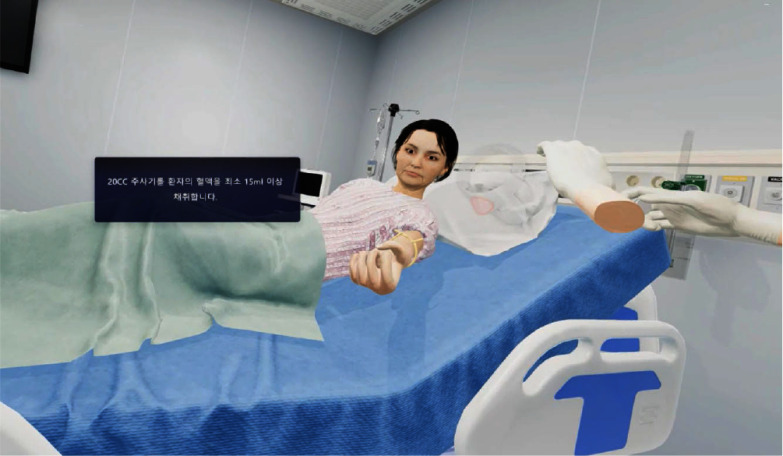

**Figure:**